# A Comprehensive, Targeted NGS Approach to Assessing Molecular Diagnosis of Lysosomal Storage Diseases

**DOI:** 10.3390/genes12111750

**Published:** 2021-10-30

**Authors:** Valentina La Cognata, Sebastiano Cavallaro

**Affiliations:** Institute for Biomedical Research and Innovation (IRIB), National Research Council (CNR), 95126 Catania, Italy; valentina.lacognata@cnr.it

**Keywords:** lysosomal storage disease (LSDs), diagnosis, targeted next generation sequencing (tNGS)

## Abstract

With over 60 different disorders and a combined incidence occurring in 1:5000–7000 live births, lysosomal storage diseases (LSDs) represent a major public health problem and constitute an enormous burden for affected individuals and their families. Several reasons make the diagnosis of LSDs an arduous task for clinicians, including the phenotype and penetrance variability, the shared signs and symptoms, and the uncertainties related to biochemical enzymatic assay results. Developing a powerful diagnostic tool based on next generation sequencing (NGS) technology may help reduce the delayed diagnostic process for these families, leading to better outcomes for current therapies and providing the basis for more appropriate genetic counseling. Herein, we employed a targeted NGS-based panel to scan the coding regions of 65 LSD-causative genes. A reference group sample (*n* = 26) with previously known genetic mutations was used to test and validate the entire workflow. Our approach demonstrated elevated analytical accuracy, sensitivity, and specificity. We believe the adoption of comprehensive targeted sequencing strategies into a routine diagnostic route may accelerate both the identification and management of LSDs with overlapping clinical profiles, producing a significant reduction in delayed diagnostic response with beneficial results in the treatment outcome.

## 1. Introduction

Lysosomal storage disorders (LSDs) are rare inherited diseases characterized by the accumulation of specific undegraded metabolites inside the lysosomes [1,2,3]. This over-storage is commonly caused by a deficiency or absent activity of lysosomal hydrolases or, in a few cases, by the deficit of further non-enzymatic lysosomal proteins (such as integral membrane proteins) [3]. With a combined incidence of 1 in 1500 to 7000 live births, this group of monogenic inborn errors of metabolism encompasses ~70 different entities, including sphingolipidoses, mucopolysaccharidoses, glycoproteinoses, lipid storage diseases, lipofuscinosis, lysosomal integral membrane proteins diseases, and post-translational modifications dysfunctions [4,5]. Clinical signs and symptoms may occur from the prenatal period to adulthood and may develop progressively over time, leading to a wide spectrum of disease phenotypes from mild to extremely severe forms that involve neuropathological effects, psychomotor development delay, cognitive decline, musculoskeletal abnormalities, dysmorphia, organomegaly, and seizures [6]. Both the considerable clinical variability within each disease phenotype and the overlapping symptomatology among single LSDs hamper the path for a precise diagnosis, which often involves a delay in treatment and severe consequences on patients’ quality of life and their families [4].

Current diagnostic workflows include an accurate evaluation of both medical history and clinical presentations, which lead to the formulation of suspicion of one or more LSDs, followed by biochemical analysis to quantify either the accumulated storage product or the enzymatic activity in leukocytes, fibroblasts, urine, or rehydrated dried blood spots (DBS) for newborns [7,8]. Finally, if deficient enzyme activity is detected, second-tier confirmatory biomarker tests or Sanger sequencing are performed for the suspected gene. Although this diagnostic route represents the current gold standard, it presents several limitations. First, it requires deep clinical expertise to discriminate phenotypic overlapping manifestations and, thus, to reduce the number of biochemical tests used for each LSD-suspected patient. Second, the execution of multiple biochemical enzymatic assays may be expensive, time-consuming, and subject to high variability, and enzymatic tests may not be available for all diseases. Therefore, reaching a definitive molecular diagnosis for LSDs with traditional techniques is still challenging, can take several years, or may be unsuccessful.

In the past decade, the emergence of next generation sequencing (NGS) technologies has proven to be an effective alternative to conventional techniques, in both research and clinical settings, allowing for the simultaneous interrogation of several genes in one single reaction and reducing, considerably, the time and costs for Sanger sequencing of a single gene [9,10]. The introduction of ad hoc designed genetic tests (targeted NGS panels) into diagnostic workflows offers the opportunity for easier identification of LSDs, timely diagnosis, and optimized clinical management, reducing the psychological burden and providing appropriate genetic counseling to parents [4].

In this study, we aimed to design and evaluate both the diagnostic utility of a semi-automated and comprehensive sequencing assay based on a targeted NGS (tNGS) panel (hereafter referred to as LSDs_panel) developed to detect pathogenic variants in 65 LSD-related genes. We describe the panel performance, strengths, and limitations and propose it as a useful second-tier diagnostic test for specialists in everyday clinical management who might suspect an LSD, given its ability to provide accurate and timely information.

## 2. Materials and Methods

### 2.1. Sample Collection and Dosage

A reference group of DNA samples isolated from clinically diagnosed donor subjects (*n* = 26) were obtained from the NIGMS Human Genetic Cell Repository at the Coriell Institute for Medical Research (https://www.coriell.org/, accessed on 26 October 2021). The purchased samples were chosen for known variants localized in targeted genes and selected in order to ensure an adequate representation for most LSDs. Quantification of the genomic DNA was assessed by measuring the genomic copies of the human *RNase P* gene using the TaqMan^®^ RNase P Detection Reagents Kit (Thermo Fisher Scientific, Waltham, MA, USA) and the Aria Dx Real-Time PCR System (Agilent Technologies, Santa Clara, CA, USA).

### 2.2. Panel Design and Library Preparation

For the selection of genes (*n* = 65) included in the panel, we relied on updated literature data [2] and a previous gene-set used for targeted strategies (Table 1). An on-demand panel (IAD199901) and a compatible made-to-order spike-in panel (IAD199905 including *TPP1* and *BLOC1S3* genes) were designed using the Ion AmpliSeq Designer software (https://ampliseq.com, accessed on 1 May 2020, Thermo Fisher Scientific, Waltham, MA, USA). The advantage of using Ion AmpliSeq on-demand panel customization is that primer pairs are pre-tested and optimized for high performance, whereas spike-ins are high concentrated made-to-order panels used to extend panels for genes not available on-demand.

The complete panel design (called LSDs_panel) covers 237.782 kb and includes 1241 amplicons with a size range of 125–275 bp distributed across two primer pools (625 primer pool 1 and 616 primer pool 2). The in silico coverage consisted of 99% for the on-demand panel and 99.18% for the spike-ins. The complete design of the LSDs_panel is available in Supplementary Table S1.

Library preparation was carried out using the Ion AmpliSeq™ Kit for Chef DL8 (DNA to Library, 8 samples/run) used for automated library preparation of the Ion AmpliSeq™ libraries on the Ion Chef™ System (Thermo Fisher Scientific, Waltham, MA, USA). According to the recommended number of amplification cycles in the standard protocol, the amplification conditions were set out to 16 cycles and four minutes of annealing/extension time. The library quality and molarity were assessed using the Ion Library TaqMan^®^ Quantitation Kit (Thermo Fisher Scientific, Waltham, MA, USA) on the Aria Dx Real-Time PCR System (Agilent Technologies, Santa Clara, CA, USA). Serial dilutions of the *E. coli DH10B* Control Library (Thermo Fisher Scientific, Waltham, MA, USA) were prepared and run in triplicate to generate a standard curve. The molar concentration of libraries was determined using the Delta R—baseline-corrected raw fluorescence calculated with Aria DX Real-Time PCR Software (Agilent Technologies, Santa Clara, CA, USA). Barcoded libraries (up to 4-Chef runs corresponding to 32 libraries) were super-pooled in equimolar concentration using the strategies suggested for combining libraries prepared with different panels for equal coverage in order to obtain a final molarity of 40 pM each.

### 2.3. Chip Loading and Sequencing

Loading of the Ion 510 and the 540 Chips was carried out using the Ion 510, 520, 530, and 540 Kit-Chef (Thermo Fisher Scientific, Waltham, MA, USA) following manufacturer instructions. High throughput sequencing runs were carried out on the Ion Gene Studio S5 system (Thermo Fisher Scientific, Waltham, MA, USA). A run planned in the S5 Torrent Suite (v. 5.12.2) had the following parameters: analysis parameters, default; reference library, hg19; target regions, LSDs_panel; read length, 200 bp; flows, 550; and base calibration mode, default. The plugins used were Coverage Analysis, Ion Reporter Uploader, and Variant Caller (default settings).

### 2.4. Variant Calling and Prioritization

Read mapping was performed automatically in Torrent Suite (v. 5.12.2, Thermo Fisher Scientific, Waltham, MA, USA) by using the variant Caller plugin (v5.12.0.4) with default settings (germline_low_stringency). The called variants were automatically uploaded on Ion Reporter (Thermo Fisher Scientific, Waltham, MA, USA). The Copy Number Variation (CNV) performance was not assessed. The pipeline analysis for variant filtering was based on multiple adjusted steps including coverage min 30×, Homopolymer length ≤ 3, *p*-value < 0.001, ClinVar ≠ benign or likely benign, MAF < 0.001 or n.a., frequency 30–60% for heterozygous variants and >70% for homozygous variants, intronic variants included if the distance from exon is < 10 bp, SIFT score < 0.05/PolyPhen score > 0.85 or n.a., and variants effect ≠ synonymous unless they are pathogenic/likely pathogenic or with conflicting interpretation of pathogenicity. A comparison of the Torrent Variant Caller (TVC) prioritized variants with their respective genetic information from Coriell biobank was performed post-analysis. True-positive (TP), true-negatives (TN), false-positive (FP), and false-negative (FN) variant calls were defined by considering available data from the single causative gene in the Coriell repository. True positives (TPs) were defined as variants both detected by our filtering pipeline as well as expected from the Coriell collected data. True negatives (TNs) were considered additional variants detected in the causative gene but excluded by our prioritization pipeline and not reported in the repository data. False positives (FPs) were considered variants detected by our pipeline but not expected from the data. False negatives (FNs) were considered variants expected from the Coriell data but missed by our pipeline. Accuracy was calculated as follows: (TP + TN)/(TP + FP + TN + FN); sensitivity was calculated as follows: TP/(TP + FN); and specificity was calculated as follows: TN/(TN + FP). The Matthews correlation coefficient (MCC) (which measures the correlation between the predicted and observed binary classification of a sample) was calculated as follows: MCC = [(TP × TN) − (FP × FN)]/√[(TP + FP)(TP + FN)(TN + FP)(TN + FN)].

## 3. Results

### 3.1. Panel Design and Performance

The LSDs_panel was designed to target the entire coding regions of 65 LSD-related genes (Table 1), which were previously reported to be a direct cause of an LSD when mutated in both alleles, in order to use it for diagnostic testing in patients with a high a priori probability of LSD based on the clinical phenotype. The LSDs_panel included 1241 amplicons (with a length of 125–275 bp) distributed between two primer pools (625 + 616 primer pairs) and covering a size of 237.782 kb, with an in silico coverage of 99% (the complete design of LSDs_panel is available in Supplementary Table S1). No additional intronic regions were targeted to maximize the coverage of exonic regions and to facilitate rapid and unambiguous interpretation in the context of diagnosis.

Before investigating the clinical utility of the gene panel, we sought to determine the analytical performance of our method in terms of depth of coverage across all targeted genes. Therefore, we used a reference group of DNA samples (*n* = 26, Table 2), isolated from clinically diagnosed donors from the NIGMS Human Genetic Cell Repository at the Coriell Institute for Medical Research and previously Sanger-sequenced for the LSD-suspected genes.

**Figure 1:**
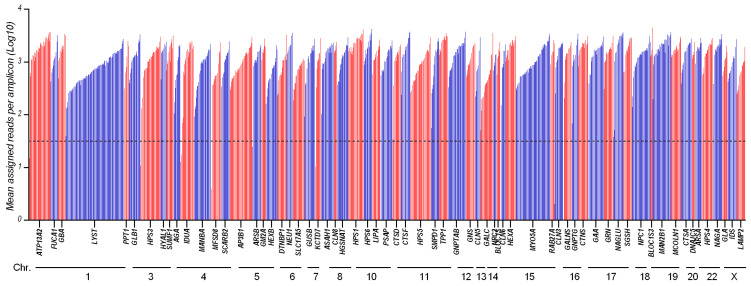
Amplicon coverage of the 65 targeted genes: 1241 amplicons distributed across 65 genes were amplified and sequenced with LSDs_panel. This chart shows the mean coverage of individual targeted amplicons across each gene for 26 analyzed samples. Amplicons with zero reads were arbitrarily represented as 0.

From the run metrics results, all samples were uniformly covered at depths that exceed the minimum coverage required (30×) for the accurate calling of variants. Coverage analysis shows that 1225/1241 of the amplicons (98.7%) had a sufficient amplification efficiency (mean assigned reads per amplicon Log10 ranging from 1.5 to 3.8), while 16 amplicons were suboptimal (Figure 1 and Supplementary Table S2).

Filtering the pipeline on the TVC (Torrent Variant Caller, Thermo Fisher Scientific, Waltham, MA, USA) was based on a stepwise-adjusted strategy to highlight relevant variants (i.e., coverage min 30×, homopolymer length ≤ 3, *p*-value < 0.001, ClinVar ≠ benign or likely benign, MAF < 0.001 or none, frequency 30–60% for heterozygous variants and >70% for homozygous variants, include intronic variants if the distance from exon is <10 bp, SIFT score < 0.05 or none, PolyPhen score > 0.85 or none, and variants effect ≠ synonymous unless they are pathogenic/likely pathogenic/uncertain significance or with conflicting interpretation of pathogenicity). A comparison with the previously known variants reported in Coriell biobank was performed by post-filtering analysis. True-positive (TP), true-negative (TN), false-positive (FP), and false-negative (FN) variant calls were defined by considering the available data from a single causative gene in the Coriell repository (see Section 2).

The overall accuracy of the panel was 98.4%, analytical sensitivity was 95.2%, while specificity was 97.6%. There were 40 correctly called true-positive variants, 83 true-negative reference calls, and 2 false-negative (missed) calls when comparing our results with the expected variants (Table 2). The MCC was 0.964 (MCC = +1 describes a perfect prediction, =0 means unable to return any valid information, and =−1 describes complete inconsistency between prediction and observation).

### 3.2. Control Samples Analysis

The majority of detected pathogenic mutations and polymorphisms are consistent with the data reported in the Coriell biobank. Interestingly, some additional observations in single causative genes emerged that are worthy to be mentioned in order to update data in the repository, as we describe below.

The NA06110 sample, acquired from Coriell biobank, derives from a female donor subject described as a compound heterozygote, with one allele carrying a G>A transition in the *SGSH* gene causing the Arg245His (R245H) aminoacidic variation and “no changes detected in the other allele”. The LSDs_panel was able to successfully detect the R245H change, identifying a second heterozygous mutation (i.e., the c.629G>A, causing the nonsense aminoacidic change—p.Trp210Ter) reported as pathogenic/likely pathogenic in ClinVar (Table 2). Thus, in addition to confirming the previously detected variant, our analysis indicated the presence of another, extending the genotypic portrait of the sample.

An additional observation is with regard to the NA02057 DNA sample, which carries a pathogenic homozygous G-to-C transversion in the *AGA* gene, resulting in a substitution of serine for cysteine at codon 163 (Cys163Ser (C163S)). The Coriell biobank reports also a heterozygous G-to-A transition (Arg161Gln (R161Q)) in the same gene, which was detected by the LSDs_panel, but classified as benign in ClinVar.

The two false negative variants were detected in the NA00879 and NA01256 samples (Table 2). The first (c.746G>A (Arg245His [R245H])) was completely missed by sequencing, whereas the second (c.1293TGG>TAG (Trp402Ter [W402X])) was detected by the panel but excluded due to very low coverage (below the threshold of 30×). We cannot rule out that missed genetic modifications are the result of high culture passages.

The LSDs_panel detected additional non-pathogenic variants in the analyzed samples (Table 2**,** in non-bold text) that may reduce enzymatic activity and may contribute to phenotypic manifestations. Given the variability of symptom manifestations as well as the phenotypic overlapping between genetically different disorders, the presence of additional secondary variants or genetic modifiers involved in lysosomal regulation and metabolism should be considered and may help to refine genotype–phenotype correlations.

## 4. Discussion

As outlined earlier, there are many factors hampering the diagnosis of LSDs, including the phenotypic and penetrance variability, the common signs and symptoms between certain disease groups, the genetic heterogeneity, and the difficulties of biochemical diagnostics. Developing a powerful diagnostic tool could mitigate the delayed diagnostic process for affected families, leading to better outcomes for current therapies and providing the basis for more appropriate genetic counseling. Many recent reports have emphasized the high clinical utility of NGS technologies and targeted gene panels in the diagnosis of suspected LSDs and their potential to reduce diagnostic delay [11,12,13,14,15,16,17].

Herein, we proposed a tNGS panel (LSDs_panel) based on AmpliSeq technologies to simultaneously screen the coding regions of 65 genes responsible for a heterogeneous group of LSDs and aimed at evaluating its clinical utility in suspected patients. By using a set (*n* = 26) of standard samples from Coriell Institute biobank (https://www.coriell.org/, accessed on 26 October 2021), we assessed the overall accuracy of the panel (98.4%), the analytical sensitivity (95.2%), and the specificity (97.6%) of the NGS workflow. Known pathogenic mutations in the reference samples were identified with the correct homozygous/heterozygous state.

Several published papers have shown the possibility of carrying out successful NGS sequencing studies from DNA extracted from Guthrie card (DBS) fingerprints, thus taking advantage of the possibility of using the same non-invasive sampling from newborns for both biochemical and sequencing tests [18,19]. Preliminary experiments in our lab starting from DBS-isolated DNA and sequenced with the LSD panel showed adequate amplicon coverage, revealing the feasibility of the NGS approach when starting from dried samples.

A second-tier application of the comprehensive LSDs_panel may be in the field of modifier genes, complex disorders, and polygenic inheritance [15,20,21]. It is well known that patients who share the same mutations may have a different phenotypic spectrum. Thus, the effect of the primary molecular defects may be modified by the presence of additional cumulative mutations located in other genes that encode proteins involved in lysosomal pathways (Table 2). The possibility of detecting variants with uncertain significance and/or secondary findings should be, however, carefully considered in reporting the results, clearing the (probable) non-causality role of the mutation. The decision to report such mutations should always be in accordance with informed consent signed by patients.

A strong limitation of the panel is the poor ability to detect complex rearrangements and recombined genomic regions, which may all require other techniques for elucidation. CNVs, including both deletions and amplifications, may be visualized starting from NGS data by manually checking the coverage of the suspected gene: the degree of coverage of the examined region with respect to the same region in other samples of the same run could suggest the presence of a CNV in heterozygous or homozygous state. However, in both cases, different molecular techniques should be used to confirm the suspected alterations as well as to exclude potential allelic dropout events.

Taken together, we demonstrated here that an NGS-based approach for the detection of LSDs may be a valuable adjunct test along with the well-established biochemical assays. Indeed, while enzyme analysis is still the gold standard for many LSDs (characterized by enzymatic deficiency), it may not accurately identify all obligate carriers and cannot be applied to disorders caused by alterations in transport or transmembrane (non-catalytic) proteins. That a broader spectrum of diseases can be monitored in one single test significantly shortens the analysis time for complex phenotypes or when a biochemical test cannot be offered. Finally, genotype–phenotype correlations may be carefully analyzed since they may be discordant, and clinicians should be cautious when counseling families regarding prognosis.

## 5. Conclusions

NGS technology is currently offering the opportunity to improve the LSD diagnostic workflow, given its low cost, semi-automated pipeline, short processing time, and ability to simultaneously detect multiple nucleotide variants on several genes. A broader adoption of targeted NGS-based tests, such as the assessment described here, should be taken into consideration to optimize clinical management of LSDs characterized by high levels of clinical and biochemical heterogeneity.

The use of targeted NGS may represent a real and valuable strategy for providing timely and correct diagnoses, for detecting carriership status, and for ensuring genetic counseling for family planning. Moreover, molecular profiling and genomic sequencing information may prompt the design of novel therapeutic drugs targeting specific mutations, thus opening the possibility for personalized medicine. Efforts in this sense may prompt patient-oriented outcomes, may improve the quality of life of patients and their families, and may reduce both direct and indirect costs (e.g., caregivers’ services) to national health services and families.

## Figures and Tables

**Table 1:** LSD-related genes included in the panel and their associated disorders.

Gene	Cytogenetic Location	Pathology	Phenotype OMIM No.
*AGA*	4q34.3	Aspartylglucosaminuria	208400
*AP3B1*	5q14.1	Hermansky–Pudlak disease type 2	608233
*ARSA*	22q13.33	Metachromatic leukodystrophy	250100
*ARSB*	5q14.1	MPS VI, also known as Maroteaux–Lamy syndrome	253200
*ASAH1*	8p22	Farber lipogranulomatosis	228000
*ATP13A2*	1p36.13	CLN12b: Kufor–Rakeb syndrome or PARK9	606693
*BLOC1S6*	15q21.1	Hermansky–Pudlak disease type 9	614171
*BLOCS13*	19q13.32	Hermansky–Pudlak disease type 8	614077
*CLN3*	16p12.1	CLN3: Batten–Spielmeyer–Sjogren disease	204200
*CLN5*	13q22.3	CLN5: Finnish variant late infantile	256731
*CLN6*	15q23	CLN6: Lake–Cavanagh or Indian variant	601780
*CLN8*	8p23.3	CLN8: northern epilepsy, epilepsy mental retardation	600143 610003
*CTNS*	17p13.2	Cystinosis	219800
*CTSA*	20q13.12	Galactosialidosis	256540
*CTSD*	11p15.5	CLN10	610127
*CTSF*	11q13.2	CLN13	615362
*DNAJC5*	20q13.33	CLN4: Parry disease and Kufs type A and B	162350
*DTNBP1*	6p22.3	Hermansky–Pudlak disease type 7	614076
*FUCA1*	1p36.11	Fucosidosis	230000
*GAA*	17q25.3	Pompe disease	232300
*GALC*	14q31.3	Globoid cell leukodystrophy, Krabbe disease	245200
*GALNS*	16q24.3	MPS IVA, also known as Morquio syndrome A	253000
*GBA*	1q22	Gaucher disease	230800
*GLA*	Xq22.1	Fabry disease	301500
*GLB1*	3p22.3	GM1 gangliosidosis; MPS IVB, also known as Morquio syndrome B	253010
*GM2A*	5q33.1	GM2 gangliosidosis, GM2 activator deficiency	272750
*GNPTAB*	12q23.2	Mucolipidosis II α/β, I-cell disease; mucolipidosis III α/β, pseudo-Hurler polydystrophy	252500 252600
*GNPTG*	16p13.3	Mucolipidosis III γ, variant pseudo-Hurler polydystrophy	252605
*GNS*	12q14.3	MPS IIID, also known as Sanfilippo syndrome D	252940
*GRN*	17q21.31	CLN11	614706
*GUSB*	7q11.21	MPS VII, also known as Sly disease	253220
*HEXA*	15q23	GM2 gangliosidosis, Tay–Sachs disease	272800
*HEXB*	5q13.3	GM2 gangliosidosis, Sandhoff diseaseb	268800
*HGSNAT*	8p11.2-p11.1	MPS IIIC, also known as Sanfilippo syndrome C	252930
*HPS1*	10q24.2	Hermansky–Pudlak disease type 1	203300
*HPS3*	3q24	Hermansky–Pudlak disease type 3	614072
*HPS4*	22q12.1	Hermansky–Pudlak disease type 4	614073
*HPS5*	11p15.1	Hermansky–Pudlak disease type 5	614074
*HPS6*	10q24.32	Hermansky–Pudlak disease type 6	614075
*HYAL1*	3p21.31	MPS IX	601492
*IDS*	Xq28	MPS II, also known as Hunter syndrome	309900
*IDUA*	4p16.3	MPS I: Hurler syndrome	607014 607015 607016
*KCTD7*	7q11.21	CLN14	611726
*LAMP2*	Xq24	Danon disease	300257
*LIPA*	10q23.31	Acid lipase deficiency: Wolman disease and cholesterol ester storage disease	278000
*LYST*	1q42.3	Chédiak–Higashi disease	214500
*MAN2B1*	19p13.13	α-Mannosidosis	248500
*MANBA*	4q24	β-Mannosidosis	248510
*MCOLN1*	19p13.2	Mucolipidosis IV	252650
*MFSD8*	4q28.2	CLN7: Turkish variant	610951
*MYO5A*	15q21.2	Griscelli syndrome 1, also known as Elejalde syndrome	214450
*NAGA*	22q13.2	Schindler disease: type Ib, also known as infantile-onset neuroaxonal dystrophy, type IIb also known as Kanzaki disease, and type IIIb, intermediate severity	609241 609242
*NAGLU*	17q21.2	MPS IIIB, also known as Sanfilippo syndrome B	252920
*NEU1*	6p21.33	Sialidosis type I, Sialidosis type II	256550
*NPC1*	18q11.2	Niemann–Pick disease types C1	257220
*NPC2*	14q24.3	Niemann–Pick disease types C1 and C2	607625
*PPT1*	1p34.2	CLN1: Haltia–Santavuori disease and INCL	256730
*PSAP*	10q22.1	Metachromatic leukodystrophy	249900
*RAB27A*	15q21.3	Griscelli syndrome 2	607624
*SCARB2*	4q21.1	Action myoclonus-renal failure syndrome	254900
*SGSH*	17q25.3	MPS IIIA, also known as Sanfilippo syndrome A	252900
*SLC17A5*	6q13	Sialic acid storage disease	269920
*SMPD1*	11p15.4	Niemann–Pick disease types A and B	257200 607616
*SUMF1*	3p26.1	Multiple sulfatase deficiency	272200
*TPP1*	11p15.4	CLN2, also known as Jansky–Bielschowsky disease	204500

**Table 2:** Detected and missed pathogenic variants in reference samples from Coriell repository.

ID Coriell Sample	Genes	Zigosity	Transcript	Coding Amino Acid Change	Variant Effect	dbSNP	ClinVar
NA03392	***GNPTG***	**Hom**	**NM_032520.5**	**c.445delG** **p.Ala149ProfsTer13**	**frameshiftDeletion**	**rs1555451874**	**P**
NA03461	***HEXA***	**Het**	**NM_000520.6**	**c.1421+1G>C** **p.?**	**unknown**	**rs147324677**	**P**
				**c.805G>A** **p.Gly269Ser**	**missense**	**rs121907954**	**P/LP**
NA05093	***GNS***	**Hom**	**NM_002076.4**	**c.1063C>T** **p.Arg355Ter**	**nonsense**	**rs119461974**	**P**
NA00654	*GLB1*	Het	NM_000404.4	c.1032T>C p.Thr344=	synonymous	rs199927127	CIP
	***MAN2B1***	**Het**	**NM_000528.4**	**c.2248C>T** **p.Arg750Trp**	**missense**	**rs80338680**	**P**
				**c.1915C>T** **p.Gln639Ter**	**nonsense**	**rs121434332**	**P**
NA02528	*AP3B1*	Het	NM_003664.5	c.1168-9C>T p.?	unknown	rs367648410	CIP
	***MCOLN1***	**Hom**	**NM_020533.3**	**c.406-2A>G** **p.?**	**unknown**	**rs104886461**	**P**
NA01675	*MFSD8*	Het	NM_152778.3	c.590G>A p.Gly197Asp	missense	rs28544073	CIP
	***GM2A***	**Hom**	**NM_000405.5**	**c.412T>C** **p.Cys138Arg**	**missense**	**rs137852797**	**P**
NA02455	***GLB1***	**Het**	**NM_000404.4**	**c.1445G>A** **p.Arg482His**	**missense**	**rs72555391**	**P**
				**c.817_818delTGinsCT** **p.Trp273Leu**	**missense**	**rs1559401428**	**P/LP**
	*CLN6*	Het	NM_017882.3	c.821C>T p.Ala274Val	missense	rs202012876	US
NA02013	***GNPTAB***	**Het**	**NM_024312.5**	**c.3501_3502delTC** **p.Leu1168GlnfsTer5**	**frameshiftDeletion**	**rs34002892**	**P**
				**c.3233_3234insCCTA** **p.Tyr1079LeufsTer3**	**frameshiftInsertion**	**-**	**n.a.**
	*GNPTG*	Het	NM_032520.5	c.574G>C p.Glu192Gln	missense	rs749314645	US
NA02552	*GLB1*	Het	NM_000404.4	c.602G>A p.Arg201His	missense	rs189115557	P
	*HPS1*	Het	NM_000195.5	c.29G>T p.Gly10Val	missense	rs759539605	n.a.
	***NAGLU***	**Het**	**NM_000263.4**	**c.889C>T** **p.Arg297Ter**	**nonsense**	**rs104894592**	**P/LP**
				**c.1928G>A** **p.Arg643His**	**missense**	**rs104894593**	**US**
NA17881	***HPS6***	**Hom**	**NM_024747.6**	**c.1714_1717delCTGT** **p.Leu572AlafsTer40**	**frameshiftDeletion**	**rs281865113**	**P**
NA17890	*LYST*	Het	NM_000081.4	c.149G>A p.Arg50Gln	missense	rs368095341	n.a.
	***AP3B1***	**Het**	**NM_003664.5**	**c.1975G>T** **p.Glu659Ter**	**nonsense**	**rs121908907**	**P**
				**c.1525C>T** **p.Arg509Ter**	**nonsense**	**rs121908906**	**P**
NA17721	***SLC17A5***	**Hom**	**NM_012434.5**	**c.115C>T** **p.Arg39Cys**	**missense**	**rs80338794**	**P**
NA16081	*PPT1*	**Het**	**NM_000310.4**	**c.451C>T** **p.Arg151Ter**	**nonsense**	**rs137852700**	**P/LP**
				**c.236A>G** **p.Asp79Gly**	**missense**	**rs137852697**	**P**
NA13204	*DTNBP1*	Het	NM_032122.5	c.489_490insT p.Lys164Ter	nonsense	-	n.a.
	***HEXA***	**Het**	**NM_000520.6**	**c.1277_1278insTATC** **p.Tyr427IlefsTer5**	**frameshiftInsertion**	**rs387906309**	**P**
				**c.805G>A** **p.Gly269Ser**	**missense**	**rs121907954**	**P/LP**
NA18455	*MANBA*	Het	NM_005908.4	c.1442A>C p.Tyr481Ser	missense	rs764041854	n.a.
	***NPC2***	**Het**	**NM_006432.5**	**c.140G>T** **p.Cys47Phe**	**missense**	**rs1555345993**	**US**
				**c.58G>T** **p.Glu20Ter**	**nonsense**	**rs80358260**	**P**
NA20387	***TPP1***	**Het**	**NM_000391.4**	**c.622C>T** **p.Arg208Ter**	**nonsense**	**rs119455955**	**P**
				**c.509-1G>C** **p.?**	**unknown**	**rs56144125**	**P**
	*GALNS*	Het	NM_000512.5	c.858G>A p.Thr286=	synonymous	rs140299014	CIP
NA20019	***ASAH1***	**Het**	**NM_004315.6**	**c.1039G>A** **p.Asp347Asn**	**missense**	**rs1354060089**	**US**
				**c.460G>T** **p.Glu154Ter**	**nonsense**	**rs1588982399**	**LP**
	*GNPTAB*	Het	NM_024312.5	c.2708_2710delTTC p.Leu904del	nonframeshiftDeletion	rs774128798	US
NA10866	*IDUA*	Het	NM_000203.5	c.785A>G p.His262Arg	missense	rs1031451164	n.a.
	***IDS***	**Hom**	**NM_000202.8**	**c.1403G>C** **p.Arg468Pro**	**missense**	**rs113993946**	**P**
NA12928	***HPS1***	**Hom**	**NM_000195.5**	**c.1484_1485insCCCCCAGCAGGGGAGG** **p.His497GlnfsTer90**	**frameshiftInsertion**	**-**	**n.a.**
	*HPS6*	Het	NM_024747.6	c.2250G>A p.Ser750=	synonymous	rs139161525	CIP
	*MYO5A*	Het	NM_000259.3	c.3567+4C>T p.?	unknown	rs186277072	n.a.
NA06110	***SGSH***	**Het**	**NM_000199.5**	**c.734G>A** **p.Arg245His**	**missense**	**rs104894635**	**P**
		Het		c.629G>A p.Trp210Ter	nonsense	rs886041370	P/LP
NA20379	***PPT1***	**Het**	**NM_000310.4**	**c.364A>T** **p.Arg122Trp**	**missense**	**rs137852695**	**P**
				**c.125G>A** **p.Gly42Glu**	**missense**	**rs386833631**	**LP**
	*GAA*	Het	NM_001079804.3	c.525delT p.Glu176ArgfsTer45	frameshiftDeletion	rs386834235	P
NA03124	*GUSB*	Het	NM_000181.4	c.454G>A p.Asp152Asn	missense	rs149606212	US
	***NPC1***	**Het**	**NM_000271.5**	**c.3182T>C** **p.Ile1061Thr**	**missense**	**rs80358259**	**P**
				**c.1947+5G>C** **p.?**	**unknown**	**rs770321568**	**CIP**
	*ARSA*	Het	NM_001085425.3	c.698_699insC p.Gln234SerfsTer41	frameshiftInsertion	-	n.a.
NA03111	***LIPA***	**Het**	**NM_001127605.3**	**c.967_968delAG** **p.Ser323LeufsTer44**	**frameshiftDeletion**	**rs917089035**	**n.a.**
				**c.894G>A** **p.Gln298=**	**synonymous**	**rs116928232**	**P/LP**
	*GALNS*	Het	NM_000512.5	c.499T>G p.Phe167Val	missense	rs148565559	US
NA02057	***AGA***	**Het**	**NM_000027.4**	**c.488G>C** **p.Cys163Ser**	**missense**	**rs121964904**	**P**
NA00879	*BLOC1S6*	Het	NM_012388.4	c.225-2_225-1insT p.?	unknown	-	n.a.
	***SGSH***	**Het**	**NM_000199.5**	**c.1339G>A** **p.Glu447Lys**	**missense**	**rs104894639**	**P/LP**
	***SGSH***	**Second Variant not detected c.746G>A (Arg245His (R245H))**					
	*CTSA*	Het	NM_000308.4	c.263_264insG p.Cys88TrpfsTer52	frameshiftInsertion	-	n.a.
NA01256	*IDUA*	Het	NM_000203.5	c.590-7G>A p.?	unknown	rs762411583	P
		**Second Variant excluded because of very low coverage c.1293TGG>TAG (Trp402Ter (W402X))**					

P = pathogenic; LP = likely pathogenic; US = uncertain significance; CIP = conflicting interpretation of pathogenicity; n.a. = not available. True-positive variants are reported in bold, and new observed findings are reported in non-bold text.

## Data Availability

Details of the reference samples selected for the present validation can be found at https://www.coriell.org/ (accessed on 26 October 2021).

## Supplementary Materials

The following are available online at https://www.mdpi.com/article/10.3390/genes12111750/s1, Table S1: Design (bed file) of the LSD panel; Table S2: Mean reads per amplicon.
